# Zinc Oxide Nanoparticles Interplay With Physiological and Biochemical Attributes in Terminal Heat Stress Alleviation in Mungbean (*Vigna radiata* L.)

**DOI:** 10.3389/fpls.2022.842349

**Published:** 2022-02-18

**Authors:** Hafiz Abdul Kareem, Muhammad Farrukh Saleem, Sana Saleem, Shabir A. Rather, Shabir Hussain Wani, Manzer H. Siddiqui, Saud Alamri, Ritesh Kumar, Nikhil B. Gaikwad, Zhipeng Guo, Junpeng Niu, Quanzhen Wang

**Affiliations:** ^1^College of Grassland Agriculture, Northwest A&F University, Xianyang, China; ^2^Department of Agronomy, University of Agriculture, Faisalabad, Pakistan; ^3^Institute of Horticultural Sciences, University of Agriculture, Faisalabad, Pakistan; ^4^Center of Integrative Conservation, Xishuangbanna Tropical Botanical Garden, Chinese Academy of Sciences, Mengla, China; ^5^State Key Laboratory of Biocontrol and Guangdong Key Laboratory of Plant Resources, School of Life Sciences, Sun Yat-sen University, Guangzhou, China; ^6^Mountain Research Centre for Field Crops, Khudwani, Sher-e-Kashmir University of Agricultural Sciences and Technology of Kashmir, Jammu and Kashmir, India; ^7^Department of Botany and Microbiology, College of Science, King Saud University, Riyadh, Saudi Arabia; ^8^Department of Agronomy, Kansas State University, Manhattan, KS, United States; ^9^Department of Botany, Shivaji University, Kolhapur, India

**Keywords:** zinc oxide, nanoparticles, osmolytes, ICPMS, heat stress, antioxidants, mungbean

## Abstract

Gradually rising atmospheric temperature is the vital component of the environment, which is anticipated as the riskiest abiotic stress for crop growth. Nanotechnology revolutionizing the agricultural sectors, notably, zinc oxide nanoparticles (nano-ZnO) has captured intensive research interests due to their distinctive properties and numerous applications against abiotic stresses. Mungbean (*Vigna radiata* L.), being a summer crop, is grown all over the world at an optimum temperature of 28–30°C. A rise in temperature above this range, particularly during the flowering stage, can jeopardize the potential performance of the plant. Hence, an outdoor study was performed to evaluate the effect of multiple suspensions of nano-ZnO (0, 15, 30, 45, and 60 mg l^–1^) on physicochemical attributes and yield of mungbean crop under heat stress. Heat stress was induced by fine-tuning of sowing time as: S1 is the optimal sowing time having day/night temperatures <40/25°C and S2 and S3 are late sown that were above >40/25°C during the flowering stage. *In vitro* studies on Zn release from nano-ZnO by inductively coupled plasma mass spectroscopy (ICPMS) disclosed that the Zn release and particles uptake from nano-ZnO were concentration-dependent. Exogenous foliar application of nano-ZnO significantly upstreamed the production of antioxidants and osmolytes to attenuate the shocks of heat stress in S2 and S3. Likewise, nano-ZnO substantially rebated the production of reactive oxygen species in both S2 and S3 that was associated with curtailment in lipid peroxidation. Adding to that, foliar-applied nano-ZnO inflates not only the chlorophyll contents and gas exchange attributes, but also the seeds per pod (SPP) and pods per plant (PPP), which results in the better grain yield under heat stress. Thus, among all the sowing dates, S1 statistically performed better than S2 and S3, although foliar exposure of nano-ZnO boosted up mungbean performance under both the no heat and heat-induced environments. Hence, foliar application of nano-ZnO can be suggested as an efficient way to protect the crop from heat stress-mediated damages with the most negligible chances of nanoparticles delivery to environmental compartments that could be possible in case of soil application.

## Introduction

Globally, it is forecasted that by the end of the twenty-first century, the air temperature will ascend by 0.2°C per decade, resulting in inflate of 1.8–4.0°C temperature more than the existing level (IPCC, 2014). Global warming has led to climate change, causing adverse effects on plant growth, as high temperatures have detrimental results on crop production worldwide (Ohama et al., 2017). Heat stress is known to alter several morphological and physicochemical processes in plants, reducing yield and disrupting cellular homeostasis (Bita and Gerats, 2013). Heat stress can disturb cellular homeostasis due to the excess production of reactive oxygen species (ROS) (Buttar et al., 2020). Under stress conditions, a substantial increase in the concentration of hydrogen peroxide (H_2_O_2_) and malondialdehyde (MDA) manifests high membrane permeability, which eventually ends up with enhanced lipid peroxidation (Bi et al., 2016; Irshad et al., 2021). Fortunately, to cope with ROS, plants have triggered specific mechanisms to reverse the decline in growth caused by heat stress, which include: (i) enzymatic and non-enzymatic antioxidant defense system and (ii) osmoregulation by producing compatible solutes (Hasanuzzaman et al., 2013). The antioxidant enzymes, such as superoxide dismutase (SOD), peroxidase (POD), and catalase (CAT) quench, and detoxify ROS from the plant cell (Hu et al., 2012; Sharma et al., 2012). When plants are exposed to stress conditions, antioxidant enzymes perform a vital role in maintaining plant growth and development and various studies have revealed a direct association between plant thermotolerance and ROS scavenging (Hameed et al., 2012). Different environmental conditions accompany different growth stages of plants. “The suitable sowing date” is one of the most imperative factors to obtain optimum yield. Plant growth is modified to a specific environmental condition at various growth stages; thus, a suitable selection of sowing dates inflates the photosynthetic efficiency (Ali et al., 2021). In Kharif season crops, during early growth stages, the temperature reaches more than 40°C. It caused a significant reduction in seed yield due to frequent flower drops, lack of fertilization, non-viability of pollen, and catalytic deactivation of rubisco activase, leading to complete enzyme failure (Perdomo et al., 2017). Heat stress harms the retention of flowering and pod formation. The primary flower shedding occurred up to 40°C temperature in mungbean and flower drop has been reported more than 79% (Kaur et al., 2015). The reproductive stage is the most sensitive stage to heat stress, resulting in loss of flower buds and seed yield (Kaushal et al., 2013; Sharma et al., 2016). Heat stress causes degradation of chlorophyll, disturbance in electron flow, disruption of photosystem II, and attenuation in carbon fixation that collectively inhibit the process of photosynthesis (Hussain et al., 2021; Li et al., 2021).

For the stability of proteins and biological membranes, Zn plays a vital role. It also facilitates maintaining ROS assembly and scavenging, as it acts as an SOD cofactor. Zn, being a part of Cu/Zn-SOD, is present within the cytosolic and chloroplast enzymes that have a crucial role against oxidative stress (Khan et al., 2021). Zn, a necessary micronutrient for all plants, takes part in numerous physiological activities, i.e., pollen performance, biosynthesis of chlorophyll, enzymes, proteins, and metabolic processes (Singh et al., 2018). It is a key component of the enzyme carbonic anhydrase, which catalyzes the exchange of CO_2_ and HCO_3_ in the mechanism of C4 photosynthesis (Faizan et al., 2021). Recent advances in nanotechnology have revolutionized agriculture and offer immense potential in enhancing abiotic stress tolerance, mainly through upregulating plant antioxidant activities (Rana et al., 2021). Nanoparticles (NPs) are atomic or molecular aggregates having at least one dimension between 1 and 100 nm that can significantly alter their physicochemical characteristics compared to the bulk material (Rastogi et al., 2017). NPs present a proficient way in an organized fashion to spray fertilizers and pesticides with the high site-specificity due to their small particles and large ratio of surface area to volume (Prasad et al., 2012; Kah et al., 2018). NPs play a pivotal role in stressed plants, which could help the plants to tolerate the abiotic stresses (Martínez-Fernández et al., 2015; Aghdam et al., 2016; Dimkpa et al., 2017). Nano-ZnO has recently captured the vast research struggles due to its distinctive quality and multiple applications in numerous fields (Sturikova et al., 2018). It has been documented that nano-ZnO is far more efficient in augmenting the productivity and absorption of Zn because of its high surface area to volume ratio (Khan et al., 2021). Under stress, NPs increase the potential of an antioxidant defense system for scavenging the free radicals by changing the microRNA expression and regulating the numerous morphological, physiological, and metabolic processes of the plant (Siddiqi and Husen, 2016; Kambe et al., 2021).

Mungbean seems to be a promising crop because it is nutritionally sound, fixes nitrogen, overgrows, and requires less water (Samson et al., 2020). As a critical pulse crop, mungbean is anticipated to be a vital component of ameliorating and diversifying cropping systems and worldwide diets. It provides vital vitamins, minerals, and amino acids (Schafleitner et al., 2015; Vittal et al., 2018). Being an imperative leguminous summer-season crop, it is much responsive to biotic and abiotic stresses. In April and May, mungbean cultivation faces temperature >30°C, which can even go up to 45°C, particularly at the time of pod filling and flowers initiation to significantly influence the yield (Hanumantharao et al., 2016; Malaviarachchi et al., 2016; Kumari et al., 2021). Foliar application of nano-ZnO against sowing time-mediated heat stress was rarely studied in previous times. Therefore, it was hypothesized that Zn as NPs is an intelligent delivery system with a larger surface area, high sorption capacity, and slow-release kinetics to target areas, which can mitigate the deleterious effects of heat activating the physiochemical mechanisms of plants against oxidative stress. One of the suitable ways to study the effect of heat stress in field conditions is to extend the sowing dates that ensure high temperature (>40/25°C) during the reproductive stage. This also provides a fair insight to study the sensitivity of a crop against heat stress. Thus, this proposed study was planned in field conditions by adjusting different sowing dates to impose heat stress at the reproductive stage. The foliar-applied nano-ZnO was applied to study the physicochemical processes and yield attributes of mungbean crops under different sowing regimes generated heat stress.

## Materials and Methods

### Characterization of Nano-ZnO

The ZnO-NPs were obtained from Alfa Aesar (Thermo Fisher Scientific, Shanghai, China), having a size range between 20 and 30 nm, density 5.606 g cm^–3^, and 99% purity. Scanning electron microscopic (SEM) (Hitachi SU8010, Tokyo, Japan) analysis was further performed to identify nano-ZnO morphology. X-ray diffraction (XRD) was used to record the peaks patterns of nano-ZnO specimens by using a Siemens D5000 diffractometer. Additionally, a UV–visible spectrophotometer (Shimadzu, Kyoto, Japan) at a 200–800 nm wavelength range was performed to examine the intensity.

### Study Site Description and Experimental Design

**FIGURE 1:**
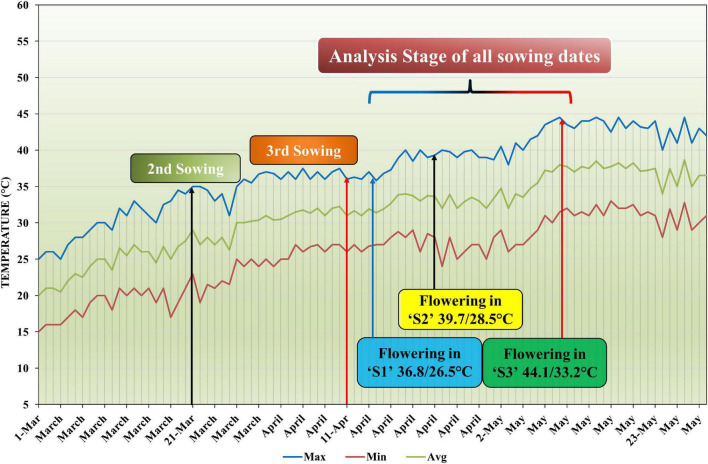
Maximum, minimum, and average temperature profile during three sowing time situations in the growing season of mungbean.

The planned research trial was conducted at the Agronomic Research Farm, University of Agriculture, Faisalabad, Pakistan in 2018 (73.06° E, 31.27° N). The climate of this region is semiarid and subtropical at an altitude of 184.4 m above sea level. The seeds of mungbean variety NM-2016 were acquired from the Nuclear Institute for Agriculture and Biology, Faisalabad, Pakistan. Mungbean seeds were treated by fungicide (azoxystrobin) and sown as a test crop in a well-prepared soil @ 20 kg ha^–1^. The layout trial of plot size 3 m × 1.8 m, comprising three replications, was used in a randomized complete block design (RCBD) under a split-plot arrangement. The chemical and physical analysis of soil was carried out by taking the soil sample earlier than sowing of the crop (Supplementary Table 1). The seedbed was prepared with the help of a cultivator followed by planking to pulverize the soil. Distance between the lines was maintained at 30 cm. Thinning was done after 10 days of sowing to maintain 10 cm distance between the plants. All the irrigations were applied according to crop requirements in all the sowing dates. First irrigation after 3 weeks of germination, second irrigation at the flowering stage, and third irrigation at pod formation were applied. Application of fertilizer was done by using the sources of urea, diammonium phosphate (DAP), and potassium sulfate (SOP) to apply the recommended dose of N:P:K at the rate of 30:60:30 kg ha^–1^ before each sowing date. Uniform agronomic operations were performed for all the treatments. Crop protection measures were adapted to save the crop from the attack of various insects, pests, and diseases. After harvesting and threshing the crop manually, seed production was counted and weighed from every plot. Grain yield (GY) was calculated as kg ha^–1^. During the mungbean growing season, data about weather details, such as daily maximum/minimum temperature, relative humidity, wind speed, and photoperiod, were obtained from the Meteorological Observatory, University of Agriculture, Faisalabad, Pakistan (Supplementary Figures 1, 2). The daily minimum and maximum temperatures were recorded and averaged each month (Figure 1).

### Zinc Oxide Nanoparticles and Heat Treatments

Heat stress was induced by setting three different sowing times, as a main plot factor, with an interval of 3 weeks: (1) the optimal sowing time S1 on 1 March 2018, when the flowering stage has day/night temperatures around 36.8/26.5°; (2) late sown S2 on 21 March 2018, having day/night temperatures 39.7/28.5°; (3) late sown S3 on 11 April 2018, when the flowering stage has day/night temperatures around 44.1/33.2° (sown in such a way that flowering stage coincided with periods of heat stress) (Figure 1). Five levels of nano-ZnO: CK (0), ZnO-1 (15 mg l^–1^), ZnO-2 (30 mg l^–1^), ZnO-3 (45 mg l^–1^), and ZnO-4 (60 mg l^–1^) were kept as subplot factor. The doses of nano-ZnO were derived by previous studies (Dhoke et al., 2011; Liu and Lal, 2015). All the solutions were prepared with deionized water. The ultrasonication of suspensions was done for 30 min to increase the dispersion of NPs. Tween 20 (0.05%) was added in solution as a surfactant to ensure the uniform retention and coverage of solution on the leaf surface. Foliar application of nano-ZnO was made at the flowering stage to study dangerous effect of the heat stress. For three replicates, the total amount of nano-ZnO used per treatment was 1 L. Each plant was sprayed three times, with the nozzle of the sprayer calibrated to deliver 1 ml of nano-ZnO in each spray.

### Biochemical Assays

After applying nano-ZnO at the flowering stage, within 24–36 h, five plants per plot were randomly selected and their entirely expanded young leaves were taken for biochemical analysis. After that, pestle and mortar were used to crush 0.5 g sample under chilled conditions at pH of 7.8. The enzyme extract was then added to 1 ml of phosphate buffer. It was centrifuged for 10 min at 4°C. After centrifugation, the residues were discarded, the supernatant was used to determine the enzymes, and the extracted material was kept at 4°C (Terras et al., 1993). Furthermore, for evaluation of enzymatic antioxidants, 96-well plates were used for pipetting of samples into it. A microplate reader was used for reading plates at different wavelengths.

### Determination of Antioxidant Enzyme Activity

For enzyme activity, the samples were prepared by grinding 0.5 g of plant leaves with 5 ml of 0.1 M potassium phosphate buffer [phosphate-buffered saline (PBS)] (pH = 7.8). Then, homogenates were centrifuged at 12,000 × g at 4°C for 20 min and the supernatants were used for the determination of the activity of antioxidant enzymes. SOD (EC, 1.15.1.1) activity was calculated at 25°C under 4,000 W (light intensity) (Giannopolitis and Ries, 1977). POD (EC, 1.11.1.7) activity was measured by using guaiacol [1-hydroxy-2-methoxybenzene (C_7_H_8_O_2_)] as an electron donor. CAT activity (EC, 1.11.1.6) was determined by measuring the conversion rate of H_2_O_2_ to water and oxygen molecules (Bianco and Defez, 2009).

### Total Phenolic Contents

Total phenolic contents (TPCs) were measured in 10 ml 80% acetone 0.5 g leaf tissue by using the Folin-Ciocalteu reagent method (Rover and Brown, 2013). After that, absorbance was read at 765 nm. The calibration curve was developed by using gallic acid (10–100 mg l^–1^) for TPC determination and results were presented as gallic acid equivalent (GAE) (Ainsworth and Gillespie, 2007).

### Gas Exchange and Chlorophyll Determinations

For the determination of gas exchange parameters [photosynthesis rate (Pn), transpiration rate (E), stomatal conductance (gs), and intracellular CO_2_ concentration (Ci)], fully expanded healthy apical leaves were used in photosynthesis apparatus Li-6400 (LI-COR Incorporation, Lincoln, Nebraska, United States). For chlorophyll determination, a 0.5 g subsample was collected of randomly taken green leaves from each experimental plot and soaked in 80% acetone overnight. 1.5 μl leaves extracts were taken to record absorbance at 663 and 645 nm in the ELISA plate. The chlorophyll a and b contents were calculated by using Eq. 1 and 2 (Arnon, 1949).


(1) $$\mathrm{Chl}\,a\,\left(\mathrm{mg}\,g-1\,\text{FW}\right)=\frac{\left[12.7\times \text{A}\,663-2.69\times \mathrm{A645}\right]\times V}{1000\times W}$$

(2) $$\mathrm{Chl}\,b\,\left(\mathrm{mg}\,g-1\,\text{FW}\right)=\frac{\left[22.9\times \mathrm{A645}-4.68\times \mathrm{A663}\right]\times V}{1000\times W}$$

Where A: absorbance, V: volume of extract (ml), and W: weight of fresh leaves tissue.

### Determination of Leaf Proline, Glycine Betaine, and Total Soluble Protein

For estimation of leaf proline (LP) content, we followed the method of Bates et al. (1973) and absorbance was recorded at 520 nm by using ELISA plates. Determination of glycine betaine (GB) was done by Grieve and Grattan (1983) method and absorbance was recorded by using an organic layer at 365 nm. Total soluble protein (TSP) was analyzed by using Bradford Reagent and were added to the ELISA plate to record absorbance at 595 nm (Bradford, 1976).

### Lipid Peroxidation (Malondialdehyde) and H_2_O_2_

The concentration of H_2_O_2_ was estimated by the method of Velikova et al. (2000). For lipid peroxidation, the content of MDA was measured by incubating tissue extract with thiobarbituric acid (TBA) at boiling temperature (Dhindsa et al., 1981).

### Yield Components

A number of pods per plant (PPP) were counted by taking five plants at random from each plot and then pods were manually measured from selected plants and then averaged. To calculate seeds per pod (SPP), five PPP were chosen randomly from each plot. In each selected plot, manually seeds were removed from every pod and averaged after counting. After manually harvesting and threshing the crop, seed production was measured and weighed from every plot. GY was calculated as kg ha^–1^.

### Inductively Coupled Plasma Mass Spectroscopy Study of Zn Dissolution

Zn release from N-ZnO was measured by inductively coupled plasma mass spectroscopy (ICPMS) (ELAN DRC-e, PerkinElmer, Waltham, Massachusetts, United States). For digestion of ultrapure HNO_3_ suspension, we followed the standard techniques of Pradhan et al. (2013), which was subsequently tested by using a Zn standard solution in an ICPMS system. All the measurements were carried out in three replicates for every treatment.

### Statistical Analysis

The collected data were statistically evaluated by using Fisher’s ANOVA and differences among treatment means were compared using the honestly significant difference (HSD) test at a 5% probability level by using Statistical Package for the Social Sciences (SPSS) software version 7 (SPSS Incorporation, Chicago, IL, United States). Graphical illustrations of statistically analyzed data were prepared by using GraphPad Prism software version 7.00 (GraphPad Software Incorporation, San Diego, California, United States). Pictorial diagrams were drawn by using Adobe Photoshop 2020. The Pearson’s correlation analyses were performed in R software version 3.6.1 to explain the correlation between plant yield and biochemical attributes with a foliar nano-ZnO application under sowing time generated heat stress.

## Results

### Characterization of Nano-ZnO

**FIGURE 2:**
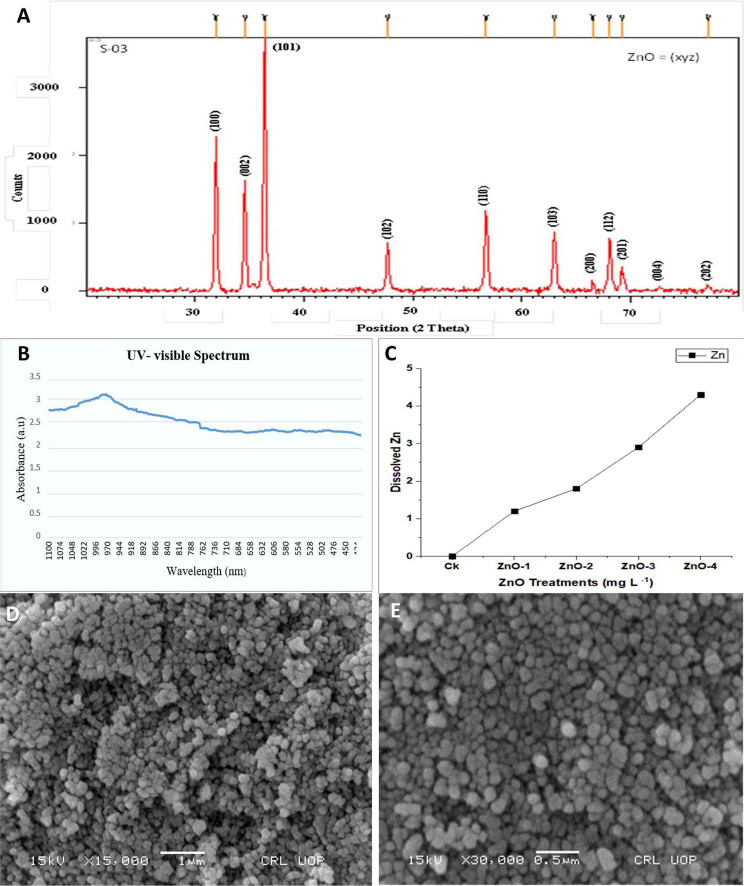
Characterization of zinc oxide nanoparticles (ZnO-NPs) by using **(A)** X-ray diffraction (XRD) spectra, **(B)** UV-visible spectrum, **(C)** nano-ZnO dissolution pattern at different concentration (CK: control, ZnO-1: 15 mg l^–1^, ZnO-2: 30 mg l^–1^, ZnO-3: 45 mg l^–1^, and ZnO-4: 45 mg l^–1^), and **(D,E)** Scanning electron microscopy (SEM).

The particle size of the sample is in the nanometer regime with an average length of 20 ± 5 nm. The XRD pattern for ZnO-NPs showed a single phase as peaks of other phases were not present and the highest peak appeared at 370 along the x-axis (Figure 2A). The UV–visible spectrophotometer results of ZnO-NPs showed that the bandgap remains almost the same (Figure 2B) as calculated by the equation:


(3) $$\text{Eg}=\frac{\text{hc}}{\lambda }$$

The surface morphology of specimen ZnO-NPs was observed near nanospheres and some nanorod shapes as shown in Figures 2D,E. These SEM, XRD, and UV–visible spectrophotometer measurements were almost the same as purchased from the company.

### Climatic Condition of the Studied Area

Daily maximum and minimum temperatures were recorded for all the sowing dates. The temperature at the flowering stage during day/night was 36.8/26.5°C for S1, 39.7/28.5°C for S2, and 44.1/33.2°C for S3 (Figure 1). Relative humidity recorded during the day/night fall between 62 and 94/20 and 52% for S1, 52 and 82/16 and 40% for S2, and 41 and 74/10 and 37% for S3 (Supplementary Figure 1). Photoperiod ranged fall between 12.8 and 13.7 in all the sowing dates.

### Antioxidant Activities

**FIGURE 3:**
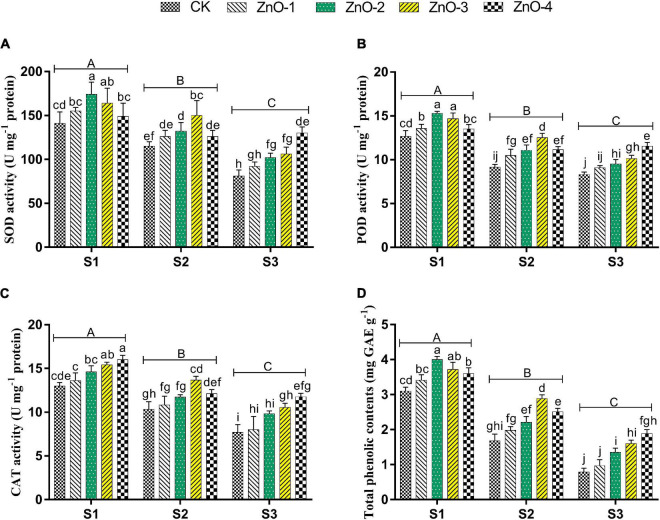
Effect of foliar application of nano-ZnO on superoxide dismutase (SOD) **(A)**, peroxidase (POD) **(B)**, catalase (CAT) **(C)**, total phenolic contents, and **(D)** in *Vigna radiata* L. under sowing time generated heat stress. Nano-ZnO doses: CK (distilled water), ZnO-1 (15 mg l^–1^), ZnO-2 (30 mg l^–1^), ZnO-3 (45 mg l^–1^), and ZnO-4 (60 mg l^–1^). S1, First sowing-March 1 (recommended); S2, Second sowing-March 22 (late sown); S3, Third sowing-April 11 (late sown). Data are mean (±SE) of three replicates and different letters designate significant changes at Tukey’s test (*p* < 0.05). In contrast, capital letters represent a significant difference among sowing time treatments and small letters represent a significant difference among the nano-ZnO treatments.

Superoxide dismutase, POD, CAT, and TPC activities were decreased under heat stress plants of S2 and S3 over ambient conditions in S1 (Figures 3A–D). In S1, the antioxidant activities, such as SOD, POD, and TPC, were increased by 21, 23, and 29%, respectively, at 30 mg l^–1^. CAT has increased by 23% at 45 mg l^–1^ doses foliar-applied nano-ZnO application compared with control. In S2, the more distant activities than control of SOD (31%), POD (37%), CAT (32%), and TPC (71%) were observed where 45 mg l^–1^ doses of nano-ZnO were applied. In S3, the activities of the enzymes, such as SOD, POD, CAT, and TPC, by 60, 39, 52, and 139%, respectively, were higher relative to control at 60 mg l^–1^ dose of foliar-applied nano-ZnO. The minimum antioxidant activities were found in control under all the three sowing times.

### Osmoprotectants and Lipid Peroxidation

**FIGURE 4:**
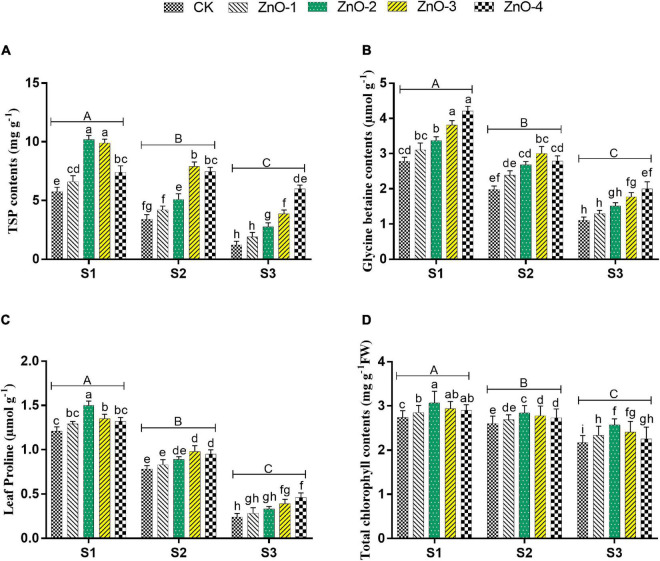
Effect of foliar application of nano-ZnO on total soluble proteins (TSPs) **(A)**, glycine betaine **(B)**, leaf proline **(C)**, and total chlorophyll contents **(D)** in *Vigna radiata* L. under sowing time generated heat stress. Nano-ZnO doses: CK (distilled water), ZnO-1 (15 mg l^–1^), ZnO-2 (30 mg l^–1^), ZnO-3 (45 mg l^–1^), and ZnO-4 (60 mg l^–1^). S1, First sowing-March 1 (recommended); S2, Second sowing-March 22 (late sown); S3, Third sowing-April 11 (late sown). Data are mean (±SE) of three replicates and different letters designate significant changes at Tukey’s test (*p* < 0.05). In contrast, capital letters represent a significant difference among sowing time treatments and small letters represent a significant difference among the nano-ZnO treatments.

**FIGURE 5:**
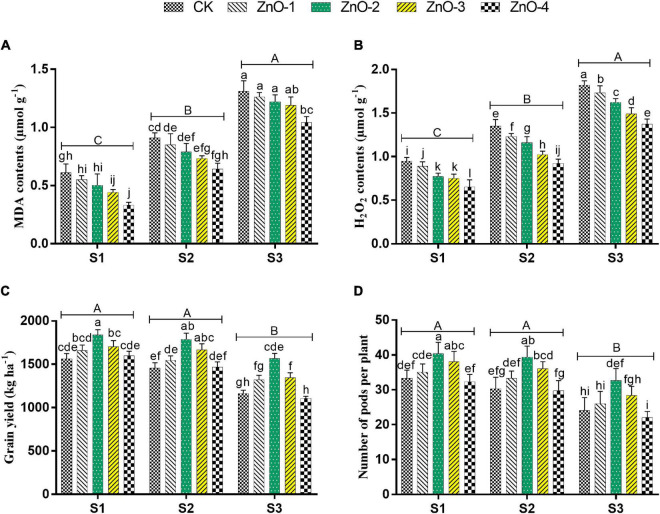
Effect of foliar application of nano-ZnO on malondialdehyde (MDA) contents **(A)**, hydrogen peroxide (H_2_O_2_) contents **(B)**, grain yield **(C)**, number of pods per plant, and **(D)** in *Vigna radiata* L. under sowing time generated heat stress. Nano-ZnO doses: CK (distilled water), ZnO-1 (15 mg l^–1^), ZnO-2 (30 mg l^–1^), ZnO-3 (45 mg l^–1^), and ZnO-4 (60 mg l^–1^). S1, First sowing-March 1 (recommended); S2, Second sowing-March 22 (late sown); S3, Third sowing-April 11 (late sown). Data are mean (±SE) of three replicates and different letters designate significant changes at Tukey’s test (*p* < 0.05). In contrast, capital letters represent a significant difference among sowing time treatments and small letters represent a significant difference among the nano-ZnO treatments.

Sowing time generated heat stress in S2 and S3 manifested maximum decrement in TSP, GB, and LP over control (Figures 4A–C), whereas conversely, the maximum increment of MDA content and H_2_O_2_ were scrutinized in S3 followed by S2 and S1, which symbolizing boosted lipid peroxidation under stressed conditions (Figures 5A,B). Under stressful environment, 60 mg l^–1^ dose of nano-ZnO significantly rebated the H_2_O_2_ and MDA contents by 25%, and 20% in S3, by 32 and 30% in S2, relative to control, respectively. In S3, the accumulation of osmolytes significantly augmented the LP, GB, and TSP by 79, 88, and 112% at 60 mg l^–1^ nano-ZnO concentration over control. Likewise, 30 and 45 mg l^–1^ levels of nano-ZnO showed better findings of osmolytes activities over control in S1 and S2, respectively. Foliar application of nano-ZnO significantly inflates the activities of osmolytes (PC, GB, and TSP) and attenuated lipid peroxidation (MDA and H_2_O_2_) compared to control among all the sowing dates.

### Gaseous Exchange and Chlorophyll Contents

Heat stress in S3 and S2 caused a significant diminution of chlorophyll contents in mungbean plants compared with S1 (Figure 4D). Heat stress-mediated higher diminishment in chlorophyll contents was marked in S3, followed by S2 and S1. Although, foliar application of nano-ZnO at 30 mg l^–1^ significantly inflates the chlorophyll contents by 14, 11, and 25% in S1, S2, and S3, respectively, compared with control among all the sowing dates. In comparison with S1, sowing time generated heat stress in S2 and S3 resulted in a significant lessening of Pn by 40 and 54%, E by 47 and 61%, gs by 31 and 48%, and Ci by 20 and 35%, respectively, whereas, foliar application of nano-ZnO at 30 mg l^–1^ resulted in the significant rise of gas exchange determinations in all the sowing dates. As, in S1: Pn by 51%, E by 87%, Gs by 58%, and Ci by 32%, in S2: Pn by 81%, E by 84%, Gs by 60%, and Ci by 41%, and in S3: Pn by 75%, E by 154%, Gs by 102%, and Ci by 34%, it gets increased relative to control (Table 1).

**TABLE 1:** Effect of foliar application of zinc oxide nanoparticles (nano-ZnO) on gas exchange determinations: photosynthetic rate (Pn), transpiration (E), stomatal conductance (gs), and intracellular CO_2_ concentration (Ci) in *Vigna radiata* (L.) under sowing time generated heat stress.

Treatments	Gas exchange attributes			
	Pn	E	gs	Ci
	(μ mol m^–2^ s^–1^)	(mmol H_2_O m^–2^ s^–1^)	(mmol H_2_O m^–2^ s^–1^)	(μ mol mol^–1^)
**1st sowing (S1)**				
Control/water spray (CK)	16.00 ± 0.57^ef^	2.55 ± 0.27^def^	0.113 ± 0.02^def^	270.3 ± 10.9^d^
15 mg L^–1^ zinc oxide (ZnO-1)	17.60 ± 0.31^cd^	3.19 ± 0.35^cd^	0.122 ± 0.02^bcd^	290.0 ± 13^cd^
30 mg L^–1^ zinc oxide (ZnO-2)	24.18 ± 0.83^a^	4.78 ± 0.33^a^	0.179 ± 0.03^a^	358.0 ± 10.2^a^
45 mg L^–1^ zinc oxide (ZnO-3)	22.11 ± 0.87^ab^	3.77 ± 0.40^b^	0.141 ± 0.01^b^	321.7 ± 12.2^b^
60 mg L^–1^ zinc oxide (ZnO-4)	20.22 ± 0.62^bc^	3.30 ± 0.23^bc^	0.134 ± 0.03^bc^	303.7 ± 9.9^c^
**2nd sowing (S2)**				
Control/water spray (CK)	9.56 ± 0.70^hi^	1.34 ± 0.35^hi^	0.078 ± 0.01^gh^	213.7 ± 15.0^ij^
15 mg L^–1^ zinc oxide (ZnO-1)	11.31 ± 0.50^hi^	1.81 ± 0.24^gh^	0.089 ± 0.01^fgh^	238.7 ± 7.9^gh^
30 mg L^–1^ zinc oxide (ZnO-2)	17.33 ± 0.60^de^	3.34 ± 0.29^c^	0.125 ± 0.02^bc^	300.7 ± 15^bc^
45 mg L^–1^ zinc oxide (ZnO-3)	15.97 ± 0.61^ef^	2.41 ± 0.18^def^	0.105 ± 0.02^ef^	263.0 ± 6.8^ef^
60 mg L^–1^ zinc oxide (ZnO-4)	14.49 ± 0.78^efg^	2.18 ± 0.26^fgh^	0.097 ± 0.02^fgh^	248.3 ± 11.7^fg^
**3rd sowing (S3)**				
Control/water spray (CK)	7.32 ± 0.46^i^	0.98 ± 0.20^i^	0.058 ± 0.01^i^	175.0 ± 8.0^k^
15 mg L^–1^ zinc oxide (ZnO-1)	8.70 ± 0.63^i^	1.44 ± 0.24^ghi^	0.071 ± 0.02^hi^	198.0 ± 14.2^jk^
30 mg L^–1^ zinc oxide (ZnO-2)	12.78 ± 0.75^fgh^	2.96 ± 0.41^de^	0.118 ± 0.01^de^	233.7 ± 8.0*h*^i^
45 mg L^–1^ zinc oxide (ZnO-3)	10.14 ± 1.02^ghi^	2.19 ± 0.23^fg^	0.099 ± 0.02^efg^	211.0 ± 15.1^ij^
60 mg L^–1^ zinc oxide (ZnO-4)	10.33 ± 0.51^hi^	1.99 ± 0.23^fgh^	0.092 ± 0.01^gh^	203.3 ± 7.2^j^

*S1, First sowing-March 1 (recommended); S2, Second sowing-March 22 (late sown); S3, Third sowing-April 11 (late sown). Data are mean (±SE) of three replicates and different letters designate significant changes at Tukey’s test (p < 0.05).*

### Yield Attributes

**FIGURE 6:**
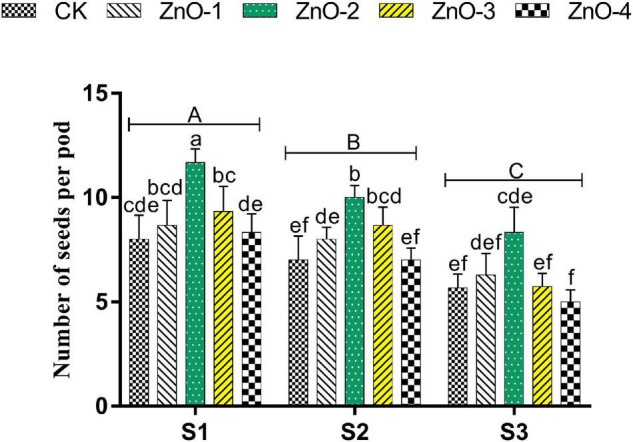
Effect of foliar application of nano-ZnO on number of seeds per pod in *Vigna radiata* under sowing time generated heat stress. Nano-ZnO doses: CK (distilled water), ZnO-1 (15 mg l^–1^), ZnO-2 (30 mg l^–1^), ZnO-3 (45 mg l^–1^), and ZnO-4 (60 mg l^–1^). S1, First sowing-March 1 (recommended); S2, Second sowing-March 22 (late sown); S3, Third sowing-April 11 (late sown). Data are mean (±SE) of three replicates and different letters designate significant changes at Tukey’s test (*p* < 0.05). In contrast, capital letters represent a significant difference among sowing time treatments and small letters represent a significant difference among the nano-ZnO treatments.

Grain yield, SPP, and PPP were significantly decreased in heat-stressed treatments S2 and S3 relative to unstressed treatment S1. Bargains in GY, SPP (Figures 5C,D), and PPP (Figure 6) levels by 18, 21, and 45% were calculated at 30 mg l^–1^ dose of nano-ZnO in S1 relative to control, respectively, whereas, foliar application of 30 mg l^–1^ nano-ZnO also significantly mitigated heat stress impact and had the highest alleviating effect in GY, SPP, and PPP by 23, 42, and 30% in S2 and 35, 48, and 37% in S3, respectively, in comparison with control.

### Inductively Coupled Plasma Mass Spectroscopy Study of Zn Release From Nano-ZnO

In *in vitro* ICPMS, after 24 h at pH 7.0, investigations on the Zn release from various concentrations of nano-ZnO of 0, 15, 30, 45, and 60 mg l^–1^ discovered that less Zn amount of 0, 1.1, 1.8, 2.9, and 4.2 mg l^–1^ was released, respectively (Figure 2C). ICPMS study revealed that Zn release and uptake from nano-ZnO were concentration-dependent.

### Correlation Between Plant Yield and Biochemical Attributes With Foliar Nano-Zno Application Under Sowing Time Generated Heat Stress

**FIGURE 7:**
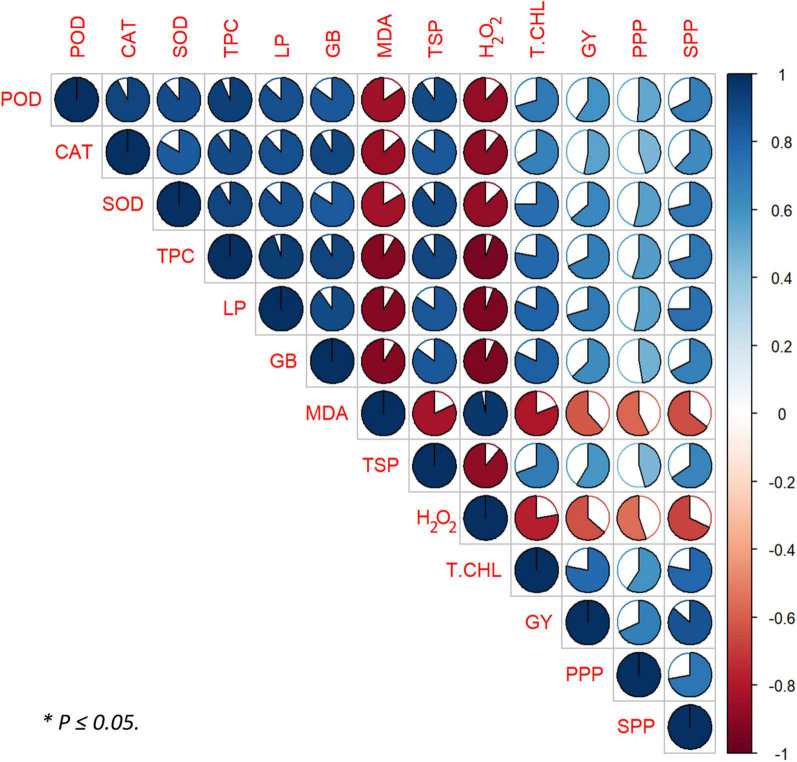
Pearson’s correlation matrix between plant yield and biochemical attributes with foliar-applied nano-ZnO under sowing time generated heat stress. Correlations are displayed in blue (positive) and red (negative); color intensity and circle size are proportional to the correlation coefficient. POD, peroxidase; CAT, catalase; SOD, superoxide dismutase; TPC, total phenolic content; LP, leaf proline; GB, glycine betaine; MDA, malondialdehyde; TSP, total soluble protein; H_2_O_2_, hydrogen peroxide; T.CHL, total chlorophyll; GY, grain yield; PPP, pods per plant; SPP, seeds per pod.

We observed a strong positive correlation between PPP, SPP, and GY being this associated with an overall plant yield obtained by foliar application of nano-ZnO under sowing time generated heat stress. GY showed a strong positive correlation with total chlorophylls, antioxidants activity, and osmolytes production and SPP and PPP in nano-ZnO sprayed plants under heat stress. Total chlorophylls contents were also positively correlated with overall antioxidant response and osmolytes activities. Plant antioxidant responses (SOD, POD, CAT, and TPC) and osmolytes (TSP, GB, and LP) pose a strong positive correlation between them. SPP and PPP also exhibit a strong positive correlation with total chlorophylls contents along with antioxidants and osmolytes activity. On the other hand, MDA and H_2_O_2_ were found to be in strong negative correlation with antioxidants activity and osmolytes production, whereas a weak negative correlation was observed with GY, PPP, and SPP (Figure 7).

## Discussion

Heat stress is an excellent debacle for the growth and productivity of crop plants. Mungbean genotype, which is used in this study, is recommended to sow on 1 March (S1); to inflict heat stress (>40/25°C) at reproductive stage, this genotype was also late sown with 3 weeks interval as S2 and S3. Subsequently, the delayed sown plants encountered the issue of heat stress at the flowering stage. The temperature was 4–6°C higher than the optimal sowing, which seriously affected the biochemical attributes performance, particularly pod set reduction, PPP, SPP, and seed yield, implying heat sensitivity of mungbean (Figure 1). Our observations on the decline in seed yield were inlined with some previous studies, which demonstrated the inhibitive effects of heat stress on mungbean crops (Sanat et al., 2006; Malaviarachchi et al., 2016).

To combat abiotic stresses, plants deploy an antioxidant defensive mechanism to ameliorate the negative influence of heat stress-generated ROS (Buttar et al., 2020). In our findings, depressed antioxidant activities were observed in heat-stressed environments S2 and S3 relative to no heat environment S1. As the level of oxidative damage is raised, the efficiency of photosynthesis decreases, which influences the growth of mungbean (Bansal et al., 2014). A direct relationship between heat stress and nano-ZnO doses was observed. Zn plays a central role in the stability of biomembranes and protein because it is a cofactor in the SOD enzyme (Faizan et al., 2020). Therefore, as the level of heat stress gets increased, the activities of SOD, POD, CAT, and TPC also get increased, at various concentrations of nano-ZnO, relative to control (Figure 3). Cu/Zn-SOD production may increase due to foliar application of nano-ZnO, which favored the defensive mechanism in scavenging of ROS, as reported by Mansoor (2013). In this study, escalation in the total phenolic activity was also examined due to the function of zinc, which coincided with the finding of Yuan et al. (2016). A strong negative correlation between antioxidants activities and MDA and H_2_O_2_ confirmed the positive role of nano-ZnO application against ROS under heat stress (Figure 7).

Osmolytes, such as LP, GB, and TSP, are low-molecular weight substances utilized by plant cells and tissues under adverse environmental conditions to keep a steady water potential by accumulating them as organic solutes to create a hypotonic environment (Yancey, 2005). Heat stress triggered the breakdown of chlorophyll that caused a reduction in sucrose synthesis, which decreased the biosynthesis of LP, GB, and TSP. Foliar applications of nano-ZnO might inflate the biosynthesis of tryptophan, which is involved in photosynthesis; hence, it increased the synthesis of sucrose required for the biosynthesis of LP, GB, and TSP (Supplementary Figure 1). Hereafter, the biosynthesis of LP, GB, and TSP was increased in S2 and S3 by exogenous foliar application of nano-ZnO under heat stress relative to control (Waraich et al., 2012). In our finding, osmolytes (LP, GB, and TSP) were found to be positively correlated with antioxidants and chlorophylls (Figure 7).

**FIGURE 8:**
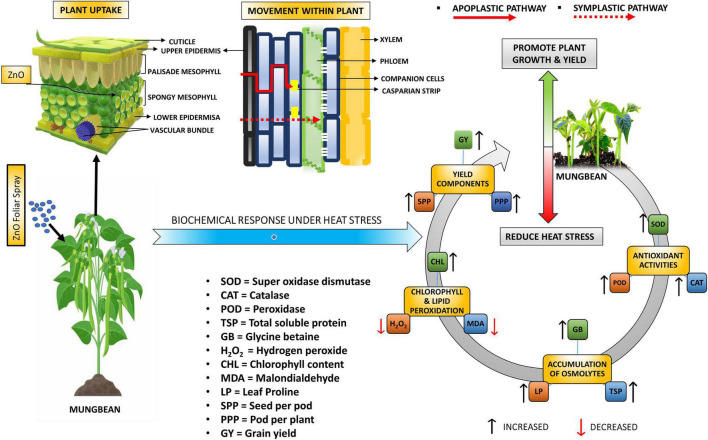
Graphical representation of plant yield and biochemical attributes with foliar-applied nano-ZnO under sowing time generated heat stress.

Peroxidation of membrane lipid is a significant damaging effect of ROS and its extent in plants is detected by measuring MDA (Garg and Manchanda, 2009; Hussain et al., 2016; Zafar et al., 2017). The curtailment in H_2_O_2_ with the escalation in foliar-applied nano-ZnO doses was noted because Zn is a part of the most abundantly found Cu/Zn-SOD in plant inflated antioxidant defense mechanism for the production of Cu/Zn-SOD to detoxify ROS (Figure 8). The findings of Disante et al. (2011) also supported the fact that under Zn deficiency, the production of Cu/Zn-SOD gets decreased, which leads to lipid peroxidation by increasing the ROS level. However, our findings showed that under S2 and S3 heat stress control conditions, the insidious effects of heat stress caused more significant lipid peroxidation by degrading biomembranes, which reduced the ability of plants to produce chlorophylls and osmolytes. Although diminution was studied in MDA contents under nano-ZnO application, Zn as a gene activator plays a crucial role in synthesizing protein and signaling. A strong negative correlation of MDA and H_2_O_2_ with antioxidants was also noted (Figure 7).

Chlorophyll is usually believed to be the most resistant pigment against heat stress. Higher temperatures eventually result in decreased photosynthesis by disrupting the function and structure of chloroplast (Faseela et al., 2020). Hence, similar findings were observed in this study, leading toward reduced chlorophyll contents (Zafar et al., 2017). Photosynthesis gets diminished due to stomatal closure as an early plant response toward heat stress, favoring better utilization of relative water contents (Tan et al., 2011). Heat stress-mediated decline in gs due to closing of stomata led to decreased Ci, which resulted in the reduction of Pn, E, pigment contents, and electron transportation to PSII (Pagter et al., 2005; Buttar et al., 2020). In this study, heat stress-mediated decrement in chlorophyll contents, Pn, E, gs, and Ci was significantly alleviated by the foliar application of nano-ZnO. As the size of applied ZnO-NPs was 20 ± 5 nm (Figure 2C), so they can easily find their way from stomatal pores to the phloem vessels *via* plasmodesmata (40 nm in diameter) and become readily available as observed by Kumar and Nagesh (2019). Hence, it was noted that nano-ZnO application potentially affected the gas exchange measures, which can be attributed to the upregulation of antioxidant defense mechanism and downregulation of ROS. Prasad et al. (2012) explicated that nano-ZnO was more effective to enhance the chlorophyll contents, flowering, and yield of pods in peanuts. Application of nano-ZnO increased the chlorophyll contents in the heat-stressed environment (S2 and S3) at specific concertation, as Zn is required for the production of chlorophyll that rebates the activity of chlorophyllase enzyme, which led to producing more chlorophyll contents in a leaf tissue as explained by Turan et al. (2009). Application of ZnO-NPs at low concentrations was found to be effective in alleviating various abiotic stresses and enhanced plant growth and development (Dhoke et al., 2011; Soliman et al., 2015). Therefore, at a higher concentration of nano-ZnO, degradation of chlorophyll led to a decrease in the overall performance of IRGA attributes. Although, the exogenous foliar applications of nano-ZnO at 30 mg l^–1^ inflated the biosynthesis of tryptophan amino acid, which is involved in photosynthesis that eventually increased the chlorophyll and growth of the plant (Waraich et al., 2012). Our findings revealed that the increased chlorophyll contents by nano-ZnO sprayed plants under heat stress was positively correlated with antioxidant enzymatic activity and osmoprotectants supporting its role in stress amelioration (Figure 7).

In contrast, earlier studies evaluating the effect of heat stress on mungbean plants grown in an outdoor environment at different intervals of time documented early shifting of phonological stages (Malaviarachchi et al., 2016). There was a phonological variation due to other sowing times in this study (Figure 1), which substantially decreased the flowering and podding duration, resulting in fewer flowers, pods, and seeds being produced. Pod set usually influenced pod and seed number, which is sensitive to heat stress in many crop species, including cotton (Snider et al., 2011), chickpea (Fang et al., 2010; Kumar et al., 2013), tomato (Li et al., 2013), and mungbean (Kaur et al., 2015).

Heat stress causes hindrance in the production of sucrose in leaves and creates an obstacle in its transport to reproductive organs (Prasad and Djanaguiraman, 2011; Li et al., 2012; Kaushal et al., 2013). Heat stress manifested significant diminution in PPP, SPP, and GY can be attributed to decreased chlorophyll contents, antioxidant activities, and osmolytes, leading to increased lipid peroxidation. The reduction in PPP and SPP caused by heat stress was reported in chickpea and soybean (Djanaguiraman et al., 2013). Many pods were aborted in heat-stressed mungbean plants of S2 and S3 as a result of limited sucrose import from the leaves. Hence, it causes a significant decrement in the number of filled pods as well as an increment in the number of infertile pods. Rainey and Griffiths (2005) demonstrated that heat stress in summers reduced yield because of pods and flowers abortion. Pod and seed diameters (data not given) were also significantly lower in S2 and S3 than in S1, indicating reduced sucrose translocation for developing sinks in heat-stressed S2 and S3 plants, as observed in chickpea (Awasthi et al., 2014) and tomato (Li et al., 2013). Although, under heat stress plants of S2 and S3, foliar-applied nano-ZnO increased PPP, SPP, and GY than control. This might be because Zn being an NPs, easily gets transported from the application site to the site of action through microcellular spaces, likely *via* plasmodesmata (Etxeberria et al., 2016). The entered nano-ZnO enhanced antioxidants competency against ROS in the plant system and produced more chlorophylls contents that ameliorate pollen performance, pollen germinability, and viability. This caused more fruit setting and eventually grew more PPP and SPP (Thakur et al., 2010; Prasad et al., 2012). Thus, nano-ZnO application in S2 and S3 ameliorated the functional aspects of reproductive organs and enhanced the antioxidant and osmolytes activities. This may be a primary cause of less pod abortion and, consequently, more pods and seeds per plant led toward more GY. Moreover, yield components (GY, SPP, and PPP) strongly correlated with chlorophyll contents, antioxidant activities, and osmoprotectants (Figure 7).

## Conclusion

Among abiotic stresses, heat stress-mediated damage resulted in fluctuation and shrinkage of crop growth, development, and overall yield. Conclusively, under the umbrella of above-discussed facts and analysis, attenuation in antioxidants and osmolytes and escalation in MDA and H_2_O_2_ were witnessed during finely tuned sowing time generated heat stress imposition in S2 and S3. Antioxidant performance, osmolytes activities, and gas exchange measures improve with foliar application of nano-ZnO in all the sowing dates, which were obvious as reduced ROS accumulation. Upregulated antioxidants and osmoprotectants prevented oxidative damage justified the beneficial role of nano-ZnO supplementations to ameliorate tolerance mechanism against heat stress and, consequently, improved yield of mungbean crop. Particularly, 30 mg l^–1^ dose of nano-ZnO performed better in all the sowing dates and better ameliorate the negative impact of heat stress. Further study is needed to be studied at the molecular level to unleash nano-ZnO-associated pathways and gene response about tolerance mechanism of heat-stressed mungbean plant.

## Data Availability Statement

The original contributions presented in the study are included in the article/Supplementary Material, further inquiries can be directed to the corresponding author.

## Author Contributions

HAK conceived the original research plans and wrote the original draft of the manuscript. MFS and QW designed and advised the research and agreed to serve as the author responsible for contact and ensure communication. HAK and JN performed the experiments. ZG assisted in microscopic experiments. SAR, SS, and SA analyzed the data and made a figure. MHS, SAR, and RK directed the experiments and revised the manuscript. NG, SW, SAR, and QW contributed to the editing and reviewing of the manuscript. All authors contributed to the article and approved the submitted version.

## Conflict of Interest

The authors declare that the research was conducted in the absence of any commercial or financial relationships that could be construed as a potential conflict of interest.

## Publisher’s Note

All claims expressed in this article are solely those of the authors and do not necessarily represent those of their affiliated organizations, or those of the publisher, the editors and the reviewers. Any product that may be evaluated in this article, or claim that may be made by its manufacturer, is not guaranteed or endorsed by the publisher.
