# Supplementary material for: Zinc Oxide Nanoparticles Interplay With Physiological and Biochemical Attributes in Terminal Heat Stress Alleviation in Mungbean (Vigna radiata L.)
Source: Front Plant Sci. 2022 Feb 18;13:842349. doi: 10.3389/fpls.2022.842349 (PMC8895266; doi:10.3389/fpls.2022.842349)
Supplement: Supplementary file 1 [file Data_Sheet_1.docx]

Appendix A

Supplementary Data

**Zinc Oxide Nanoparticles (Nano-ZnO) Interplay with Physiological and Biochemical Attributes in Terminal Heat Stress Alleviation in Mungbean (*Vigna Radiata* L.)**

**Hafiz Abdul Kareem^1,2^, Muhammad Farrukh Saleem^2^, Sana Saleem^3^, Shabir A. Rather^4,5^, Shabir Hussain Wani^6^, Manzer H. Siddiqui^7^, Saud Alamri^7^, Ritesh Kumar^8^, Nikhil B. Gaikwad^9^, Zhipeng Guo^1^, Junpeng Niu^1^ and Quanzhen Wang^1^ *****

*^1^ College of Grassland Agriculture, Northwest A&F University, Yangling, Shaanxi, 712100, China*

*^2^ Department of Agronomy, University of Agriculture, Faisalabad 38040, Pakistan*

*^3^ Institute of Horticultural Sciences, University of Agriculture, Faisalabad 38040, Pakistan*

*^4^ Center of Integrative Conservation, Xishuangbanna Tropical Botanical Garden, Chinese Academy of Sciences, Menglun, Yunnan, China*

*^5^ State Key Laboratory of Biocontrol and Guangdong Key Laboratory of Plant Resources, School of Life Sciences, Sun Yatsen University, Guangzhou 510275, China*

*^6^ Mountain Research Centre for Field Crops, Khudwani, Anantnag-192 101, Sher-e-Kashmir University of Agricultural Sciences and Technology of Kashmir, J&K, India.*

*^7^Department of Botany and Microbiology, College of Science, King Saud University, Riyadh, 11451, Saudi Arabia.*

*^8^ Department of Agronomy, Kansas State University, Manhattan, KS, 66506, USA*

*^9^ Department of Botany, Shivaji University Kolhapur, India-416004*

** Correspondence:* [*wangquanzhen191@163.com*](mailto:wangquanzhen191@163.com)

**Corresponding author:**

Quanzhen, Wang

Taicheng Road, No. 3, Yangling-712100 Shaanxi, China.

Tel: +86-13759942845

E-mail: [wangquanzhen191@nwsuaf.edu.cn](mailto:wangquanzhen191@nwsuaf.edu.cn)

**Table 1S: Chemical analysis of air-dried soil before crop sowing.**

| **Characteristics.** | **Unit.** | **Value. obtained** | **Status.** |
| --- | --- | --- | --- |
| Texture. | _ | _ | Sandy loam. |
| pH. | _ | 8.2. ± 0.75 | Alkaline. |
| Sand. | %. | 33.18. ± 3.12 | _ |
| Silt. | %. | 34.12. ± 2.65 | _ |
| Clay. | %. | 32.31 ± 1.15. | _ |
| EC. | .dS m^-1^ | 1.2. ± 0.67 | Normal. |
| Organic matter. | %. | .0.92 ± 0.26 | Low. |
| Nitrogen (N). | %. | .0.058 ± 0.02 | Low. |
| Phosphorus (P). | Ppm | 8.9 ± 0.95 | Low. |
| Potassium (K). | Ppm | 166 ± 6.7 | Sufficient. |
| Zinc (Zn) | Ppm | 0.60 ± 0.20 | Low. |
| Boron (B) | Ppm | 0.49 ± 0.16 | Low. |

**Fig. 1S.** Relative humidity during all sowing time situations in growing season of mungbean


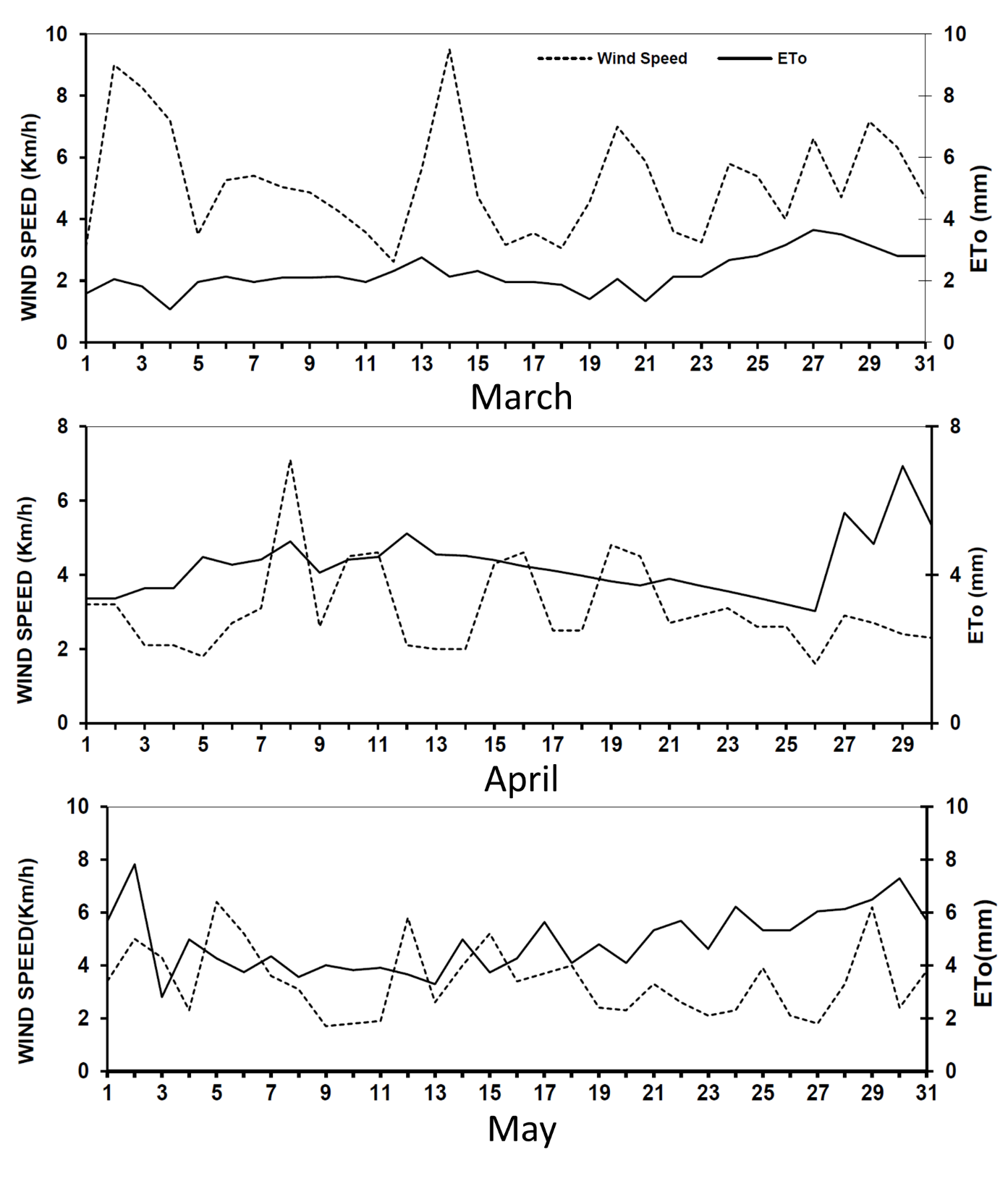
**Fig. 2S.** Wind speed and ETo during all sowing time situations in growing season of mungbean
